# An analysis of the generalization of pretend play from real objects to toys

**DOI:** 10.1002/jaba.70017

**Published:** 2025-06-10

**Authors:** Toni Rose T. Agana, Tina M. Sidener, Nicole M. Rodriguez, Sharon A. Reeve, Heather M. Pane

**Affiliations:** ^1^ Munroe‐Meyer Institute University of Nebraska Medical Center Omaha NE USA; ^2^ Department of Applied Behavior Analysis Caldwell University Caldwell NJ USA; ^3^ Department of Behavioral Science Daemen University Amherst NY USA

**Keywords:** autism, generalization, learned‐combinations play, pretend play, toys

## Abstract

Researchers have reported that children engage in pretend play that reflects the conventional activities of their environment (i.e., *learned‐combinations play*). In contrast, children with autism spectrum disorder (ASD) display fewer and less varied play behaviors. Research on teaching pretend play to children with ASD often involves prompting and reinforcing scripted responses. Although effective, these procedures may be limited because they can produce rigid, rote play rather than pretend play reenacting real‐life events. This study evaluated the effects of teaching actions with real objects on the emergence of generalized learned‐combinations play with three children with ASD. Overall, teaching children actions using real objects facilitated generalization of those actions with toys. We provide future directions for research when limited generalization is observed with some toys. Additionally, we discuss the implications of automatic reinforcement and the motivating operation in relation to our findings.

Cognitive‐developmental psychologist, Jean Piaget, famously characterized pretend play as the “happy display of known actions” (1962, p. 93). Expanding on Piaget's perspective, recent research in developmental psychology (e.g., Lifter et al., 2022) has shown that certain pretend play actions of neurotypical children represent conventional activities of their environment. That is, children reenact with toys (e.g., pretending to smell a toy flower) activities they have experienced in the past (e.g., smelling a real flower; Fein, 1981; Lillard et al., 2013; Lockman & Tamis‐LeMonda, 2021), which we refer to as *learned‐combinations play* (Lifter et al., 2022; Unhjem et al., 2014).

Bijou's (1995) analysis of child development from a natural science perspective may offer a broader framework for understanding the mechanisms underlying learned‐combinations play. Bijou emphasized that a substantial part of child development involves responding coming under control of discriminative stimuli and generalization across varying contexts. He noted that when a stimulus signals the availability of reinforcement, other similar stimuli may also serve as discriminative stimuli. This could explain how children transition from interacting with real objects to engaging in play actions with toys that share perceptual similarities. Thus, learned‐combinations play may be understood as a natural outcome of stimulus generalization in which experiences with real‐world objects subsequently occasion interactions with toys in ways that reflect everyday activities.

Studies have found that children diagnosed with autism spectrum disorder (ASD) engage in fewer pretend play actions than their neurotypical peers (e.g., Wilson et al., 2017); however, it is unclear whether the kind of pretend play referred to in these studies included learned combinations or other types of pretend play (e.g., using an object to stand for another, adopting a role that does not belong to the child; Lang et al., 2009; Lifter et al., 2022). Several interventions have demonstrated efficacy in establishing pretend play with children with ASD (Stahmer et al., 2003). These interventions typically employ strategies to prompt play actions such as physical guidance (Groskreutz et al., 2011) and modeling (Scheflen et al., 2012). Additionally, contrived consequences (e.g., praising placing a peg in a board) rather than natural consequences that are not socially mediated (e.g., the peg standing up after being placed in a board) are common treatment components for teaching children with ASD to play (Barton & Wolery, 2008; Carmody & Stauch, 2022; Lee et al., 2020). These procedures, although effective, may be limited in teaching a child with ASD to engage in pretend play of reenacting real‐life events and instead may produce rigid, rote play (Luckett et al., 2007). If these play behaviors are not maintained by the natural consequences in play environments, they are unlikely to continue in the absence of contrived consequences.

Although few studies have evaluated methods for teaching play in the absence of prompts and contrived consequences, a recent study by Lee et al. (2021) evaluated an intervention for teaching children with ASD to engage in pretend tacts with toys by first teaching them to tact different stimulus properties of real objects. For example, participants tasted an apple and said, “It tastes sweet” when presented with a toy apple and the question, “How does it taste?” The general intervention involved multiple‐exemplar training and presenting real objects (e.g., real apple) for participants to experience their stimulus properties (e.g., tasting the apple). Correct responses were praised, and the child was allowed to consume or engage with the real object. If the child did not emit the target response, the experimenter directed the child to interact with the real object (e.g., “Taste it and let me know how it tastes”). These procedures resulted in generalization of tacts of stimulus properties from real objects to toys across all three participants, aligning with Bijou's (1995) framework on the emergence of generalization in child development.

There are some considerations and limitations of Lee et al. (2021) worth noting. First, Lee et al. discussed that using reinforcement during probes with the toys may limit the interpretation of the results as generalization. Second, the study focused on play related to verbal behavior, without addressing nonverbal play behavior, which is an important part of play. Finally, although multiple‐exemplar training may have produced generalization of tact responses to play stimuli, using multiple exemplars may not always be successful in producing generalized responses (Engelmann & Carnine, 1982; Holth, 2017). Because the resemblance of play stimuli to real objects may contribute to the generalization of tact responses, careful selection of teaching examples may be useful.

General‐case procedures refer to a systematic method to program for generalization by selecting sufficient exemplars across a full range of both trained and untrained exemplars to achieve correct performance (Horner et al., 1984). Within these procedures, general‐case analysis involves developing appropriate sources of stimulus control by identifying necessary responses in natural settings, along with identifying the critical and noncritical features present when those responses occur (Horner et al., 1984). To our knowledge, only one study has employed general‐case procedures to teach toy play to children with ASD. Haring (1985) evaluated the effects of generalization training (e.g., using general‐case procedures) with four children with ASD, using four sets of toys to assess generalization to untrained toys. Specifically, the construction of these toy sets followed the general‐case procedures outlined by Horner et al. (1984). Moreover, the toys in each set were carefully selected to include the critical features (e.g., toy airplane included a long body and a wing on each side of the body), evolving progressively from specific to more abstract characteristics. For example, the least abstract toy airplane in the set was a model of a 747 jet, whereas the most abstract toy comprised two Lincoln logs intersecting at right angles and secured with tape. These procedures resulted in generalization during probes with untrained toys without prompts or praise.

The purpose of this study was to evaluate the efficacy of teaching actions with real objects on learned combinations of play actions using toys that were perceptually similar to the real objects with three children with ASD. Specifically, this study addressed previous research by (a) evaluating the development of learned‐combinations play actions, (b) ensuring no contrived consequences occurred during toy probes, and (c) using general‐case analysis, by extending Haring (1985), to identify and create exemplars of toys and real objects of specific activities.

## METHOD

### Participants and setting

Participants included three children (two female, one male) diagnosed with ASD by an independent specialist prior to the study. Participants qualified for the study if they demonstrated the following skills during preexperimental assessments: (a) demonstrating perceptual similarity‐based matching, (b) imitating actions with objects, and (c) obtaining scores on the Developmental Play Assessment for Practitioners (Lifter et al., 2022; see *Developmental play assessment* section for details) that demonstrated emerging or mastery skills in Level 6, *Learned Combinations of Objects*. Sessions were conducted in a university clinic that took place in either an individual session room or a setting that was tailored for a particular activity (e.g., a kitchen for making s'mores).

Heidi (8 years, 4 months; female; Asian, White, and Hispanic) began receiving early intensive behavioral intervention services when she was 5 years old. She scored 165 points on the Verbal Behavior Milestones Assessment and Placement Program (VB‐MAPP; Sundberg, 2008) with mastery of skills in Levels 1 and 2 and approaching mastery of Level 3. Heidi demonstrated advanced verbal behavior skills, her vocalizations were intelligible, and she used accurate sentence structures when talking to others. Additionally, at the beginning of the study, Heidi was in the process of transitioning from an early intervention program to a social skills program. Her mother reported that Heidi engaged in some pretend play, specifically moving figures capable of actions and enjoyed playing with small toy figures such as Peppa Pig.

Roy (7 years, 6 months; male; White) began receiving early intensive behavioral intervention services when he was 3 years old. He scored 150.5 points on the VB‐MAPP with mastery of skills in Level 1 and approaching mastery of Levels 2 and 3. Roy demonstrated advanced verbal behavior skills, his vocalizations were intelligible, and his sentence structures were accurate when talking to others. Additionally, Roy would soon be transitioning from an early intervention program to a social skills program. Prior to the study, Roy's mother reported that Roy engaged in minimal pretend play actions, primarily limited to dinosaurs. Roy also enjoyed constructing toys (e.g., marble tower) and showed preference for playing independently.

Belle (8 years, 11 months; female; White) began receiving early intensive behavioral intervention services when she was 4 years old. She scored 145 points on the VB‐MAPP with mastery of skills in Level 1 and approaching mastery of Levels 2 and 3. Belle emitted simple sentence structures (e.g., “I want iPad,” “chase me”), and her vocalizations were not consistently intelligible enough to be understood by others without context. Belle's caregivers reported that she rarely engaged in toy play other than lining up toy figures. Instead, Belle spent most of her leisure time watching videos on a device, coloring, and drawing.

### Preexperimental procedures

#### Imitation skills assessment

The Motor Imitation Scale (Stone et al., 1997) was administered with each participant to ensure that they could imitate motor actions with objects. The Motor Imitation Scale is a 16‐item tool used to assess motor imitation skills for young children with ASD. Half of the actions require imitation with objects, and the other half require imitation of body movements without objects. Only actions with objects from the Motor Imitation Scale were presented, and each action with objects was presented three times. The participant's response to each action was scored from 0 to 2. Eligibility criterion was a score of 2 for each action (i.e., total of 16); all participants met the eligibility criterion.

#### Perceptual similarity‐based matching assessment

The purpose of this assessment was to determine whether the participant could match a class of stimuli with common characteristics (Fields & Reeve, 2000). Specifically, the participants were asked to match a toy to the corresponding object. The assessment consisted of 10 targets that were different from the play targets of the study. Toys available at the clinic setting were selected as the items for matching. During this assessment, the comparison stimuli were presented in an array of three items (e.g., hairbrush, bowl, car), and the participants were asked to “match” when given the sample stimulus of an item (e.g., toy hairbrush). Ten trials were presented across two sessions. All participants scored 100%.

#### Developmental play assessment

The Developmental Play Assessment for Practitioners (Lifter et al., 2022) was administered with each participant. It is an assessment based on the developmental progression of play by children, as proposed by Lifter and Bloom (1989). Per the manual instructions, direct observations were conducted with each participant and data were collected on the frequency of play actions in each play category and the frequency of play exemplars the participant demonstrated. As recommended by Lifter et al. (2022), the emerging developmental status (i.e., at least two occurrences of responses in the category, with two different exemplars) was selected for intervention. The assessment results indicated that participants met the inclusion criteria by demonstrating mastery of Level 6, Learned Combinations of Objects, which involves a single play action that represents conventional activities of the culture (e.g., smelling a flower). However, to align with the participants' emerging developmental status, the selected developmental play category was Level 9, Varied Action Sequences, which involves acting out two or more different play activities or actions. Furthermore, reenacting real‐life events (i.e., learned‐combinations play) was incorporated into the Varied Action Sequences play actions that corresponded to the actions taught with the real objects. An example of a play target included a toy set with flowers and a vase with the following two actions: (1) putting a flower to the nose and (2) putting the flower in the vase.

#### Materials and selection of play targets

Five play actions were targeted per participant. For three targets, the experimenter taught the actions with real objects and assessed stimulus generalization with toys, using three real objects and three toy exemplars for each. For example, the flower target included three real flowers and three toy flowers. The fourth target, taught by a therapist, involved everyday actions completed with their therapist, and the fifth was an activity the participant already engaged in at home with caregivers. The fourth and fifth play targets allowed for the assessment of across‐category generalization and included three toy exemplars each. See Table 1 for Heidi's targets and stimuli; Supporting Information A and B contain information about the targets and stimuli for Roy and Belle.

**TABLE 1:** Targets and stimuli for Heidi. The targeted play category was Varied Action Sequences, which consists of acting out two or more different play actions. N/A = Not applicable.

Stimuli, action, vocalization	Target 1	Target 2	Target 3	Across category (Therapist)	Across category (Caregiver)
Stimuli	Marshmallow, graham crackers, chocolate, stick	Flower, vase	Fish, fishbowl, fish food container	Iron, iron board, clothing item	Toothbrush, cup
Action	(1) Put marshmallow on top of chocolate (2) Put cracker on top of marshmallow	(1) Put flower to nose (2) Put flower in vase	(1) Put finger on fishbowl (2) Put fish food above fishbowl	(1) Turn dial on iron (2) Move iron side to side on clothing item	(1) Put toothbrush to teeth (2) Put cup to mouth
Vocalization	(1) “Marsh‐ mallow” (2) “Yummy!”	(1) “Smells good” (2) “So pretty”	(1) “Hi, fishie!” (2) “Eat some food”	(1) “Make it hot” (2) “Then smooth”	N/A

Target selection for play activities was determined by participant history, toy availability, feasibility of presentation, caregiver social validity reports, and dissimilarity of action topographies and materials. First, the *Family Values and Experience Questionnaire* (FaVE; Agana et al., 2024) was administered to each of the participant's caregivers. The FaVE is a 32‐item questionnaire assessing the participant's and family's preferences, values, and experiences across categories such as family structure, languages, activities, food, sleep, transportation, school, and play. Based on the FaVE results, four targets (i.e., three taught by the experimenter and one by a behavior therapist) involved activities the participant observed but did not directly engage in, whereas another target was selected based on a daily activity the participant already engaged in at home. Moreover, the feasibility of presenting the activities was considered (e.g., presenting real flowers for the participant to smell during teaching). Once the targets were selected, the caregivers were asked if they agreed with the selected targets and toys that would be used in the study.

#### General‐case analysis for exemplar selection

A general‐case analysis was conducted to identify various exemplars of the toys and real objects of a specific activity to increase the likelihood that critical features (e.g., stem, petal structure) would occasion a specific response (e.g., smelling a flower; Horner et al., 1984; Milata et al., 2020). First, targets were defined based on required responses. For example, the required response for the target smelling a flower and putting it in the vase was to make the flower touch the nose and to make the flower stem go inside the vase. Second, critical and noncritical features of the real objects were identified. For example, the critical features of a flower include a stem at the bottom and a petal structure at the top and the critical features of a vase include opening at the top and base at the bottom. The noncritical features that vary across flowers include color and size of stem, variation in the color and shape of the petal structure, presence of leaves on the stem, and the presence or absence of a scent; the noncritical features that vary across vases include color, size, design, and the presence of water inside.

Third, the critical and noncritical features were organized into categories that were used to identify exemplars across a gradient ranging from toys that closely resemble real objects to those that are less similar (Haring, 1985) as well as real objects found in natural environments. Real object exemplars were selected by combining all the critical features and variations of noncritical features of a stimulus class. For example, real flowers used included all the critical features listed above. The noncritical features that varied across the real flowers used in the study included the shade of color in the stem, variation in the shape of the petal structure, and presence of leaves on the stem. Conversely, the selection of the three toy exemplars for each play target were selected to evaluate a generalization gradient such that one toy was *most similar* to the corresponding real objects (i.e., included the most common features of the flower but were not considered as critical features such as scent and leaves), one toy was *moderately similar* to the corresponding real objects (i.e., included fewer common features), and one toy was *least similar* to the corresponding real objects (i.e., included the least common features). Each toy set (i.e., target) consisted of one toy from each of the categories stated above. Selecting toys across this gradient allowed for assessing whether participants were more likely to generalize actions learned from real objects to toys that were most or least similar to the real objects. See Table 2 for the toy exemplars used for the flower play target. All participants had the same real object and toy exemplars as well as play targets, except for one of Roy's targets (i.e., donut) because he engaged in the learned‐combinations play action with the flower target during baseline. Supporting Information C (s'more), D (fish), E (donut), F (iron), and G (toothbrush) include the additional play target exemplars.

**TABLE 2:** Flower toy exemplars. The common features were systematically selected to closely resemble the most common characteristics of real objects depicted by similar toys. Moderately and least similar toys showed fewer common features representing real objects.

Features	Stimulus	Most similar	Moderately similar	Least similar
Critical features	*Flower*: stem, petal structure *Vase*: opening or mouth, base or foot	All critical features included		
Noncritical features	*Flower*: color, size *Vase*: color, design, size	Noncritical features varied		
Common features	*Flower*: leaves, scent *Vase*: water	*Flower*: leaves, scent *Vase*: water	*Flower*: leaves *Vase*: water	*Flower*: leaves *Vase*: none

### Dependent variable and measurement

During the generalization probes with toys and teaching sessions with real objects, trial‐by‐trial data were collected on the occurrence of learned‐combinations play actions and vocalizations. A *learned‐combinations play action* was defined as a discrete play action by the participant that was previously taught by the experimenter during teaching with the real objects and resulted in the same change in the environment (i.e., same naturally occurring consequences of the response). Additionally, each *discrete play action* was defined as a motor movement with a beginning (e.g., participant takes hold of the toy flower), an action performed with the toy (e.g., bring flower to nose), and an end (e.g., participant lets go of the flower; Lifter et al., 2022). To encompass the development‐matched play target (i.e., Varied Action Sequences) identified by the Developmental Play Assessment for Practitioners, each discrete play action included a two‐sequence action. A *learned‐combinations play vocalization* was defined as a vocal response by the participant that was previously modeled by the experimenter during teaching with the real objects, except for differences in conjunctions, articles, or changes in verb tenses (e.g., “So pretty” would be scored the same as “It's so pretty”; Krantz & McClannahan, 1993).

### Experimental design and procedure

A multiple‐probe design (Horner & Baer, 1978) across participants was used to evaluate the effects of teaching actions with real objects on learned‐combinations play actions with perceptually similar toys. Baseline sessions with the toys were conducted first, followed by the teaching of actions using real objects. Generalization probes were administered during the real object teaching phase. After a participant demonstrated mastery with real objects by scoring 100% across three consecutive sessions, we analyzed the data to identify a trend (if any) with the toys. A play target was considered mastered if at least two correct responses out of three exemplars occurred across three sessions. If targets remained unmastered after real object mastery or responding was variable or decreased, the question “Can you show me what else you can do with the toys?” was introduced.

#### Baseline (with toys)

Each baseline session comprised nine trials, arranged in a quasirandomized sequence. These trials included three play targets and three exemplars for each toy set. Each trial began with the experimenter presenting a toy set (e.g., toy flower, toy vase) and saying, “Here are toys to play with.” The participant was given up to 60 s to manipulate the toys or until the participant demonstrated they were done with the toys such as saying “all done” or similar phrases, pushing or throwing the toys, leaving the play area, or absence of toy play for 15 consecutive seconds. No prompts or programmed consequences were provided. If the participant initiated an interaction with the experimenter (e.g., looked at the experimenter and said, “Flower”), then the experimenter responded with a general comment (e.g., “It is a flower”).

#### Real object baseline and teaching

Prior to teaching with real objects, baseline data were collected to observe whether the participant emitted the targeted actions. Each session included nine quasirandomized trials with three exemplars of each real object. The experimenter presented the objects (e.g., flower and vase) and said, “Here are (name of objects) for you.” The participant had 10 s to manipulate the objects or indicate they were done (e.g., saying “All done”). If the target action was emitted, the experimenter provided praise and a preferred item; if not, the experimenter initiated the next trial.

Teaching sessions with real objects followed baseline procedures with the addition of a least‐to‐most prompting hierarchy. The hierarchy included modeling of the targeted action and vocalization with the instruction, “Copy me” or a similar instruction, partial physical prompts of the action, and full physical prompts of the action. The participant was provided 3 s to emit the targeted action between prompts. Specific praise (e.g., “Nice! You smelled and put the flower in the vase!”) and a preferred item were provided for correct responses without prompts, whereas only praise was given for correct responses following a prompt. Mastery of the real object teaching was 100% correct responding across three consecutive sessions.

#### Generalization probes with toys

Generalization probes with toys were conducted in a manner similar to that for baseline (with toys) sessions to evaluate generalized play with the toys after teaching with the real objects. For example, after teaching with real objects of the flower, a probe with a toy flower set was conducted. Once mastery criterion was met for the generalization probes with toys, then intervention was complete for that specific play target.

##### Question

If variable responding or a decreasing trend was observed in the generalization probes across three consecutive sessions after mastering real objects, the question “Can you show me what else you can do with the toys?” was introduced for unmastered targets. Sets already mastered were not included in this condition. If no response or repeated action occurred within 5 s of the toy probe, the experimenter presented the question and re‐presented the toy set. No programmed consequences were provided. It should be noted that only Heidi and Roy required the question for some unmastered targets after teaching with the real objects.

#### Across‐category generalization

Across‐category generalization targets with six trials were conducted as pre‐ and posttest measures, following baseline procedures. The participant's behavior therapist taught one target (i.e., iron) daily after baseline sessions, and the other target involved an activity already occurring daily at home (i.e., toothbrush), as reported by caregivers. Additionally, it should be noted that there was not a corresponding vocalization for the toothbrush target, as we did not have information on the specific vocalizations participants encountered at home.

#### Maintenance probe

A maintenance probe was conducted 1 day after participants engaged with the real objects. The purpose of this probe was to assess whether participants would maintain the learned combinations of play actions and vocalizations after a longer interval between engaging with real objects and generalization probes with toys. The maintenance probe was conducted similarly to the baseline sessions with toys. The only difference was that the question (i.e., “Can you show me what else you can do with the toys?”) was provided if a participant did not engage in the learned combinations of play action with a toy.

### Social validity

Social validity was assessed pre‐ and postintervention. Supporting Information H includes the questionnaire. Preintervention assessment evaluated the social validity of the goals and procedures. Specifically, an interview was conducted with the caregivers to evaluate the social validity of the targets and toys and ensure that they agreed with the targets and toys used for the study. Caregivers confirmed agreement with the selected targets and toys; no adjustments were needed.

Postintervention assessment of social validity was conducted by having caregivers watch video recordings of sessions and rate their satisfaction with the study's goals, procedures, and outcomes on a 5‐point Likert scale (i.e., 1 = *strongly disagree*, 5 = *strongly agree*), along with space for comments. Three open‐ended questions about the beneficial and challenging aspects of the study procedures were also included as well as questions concerning child enjoyment during the study. The caregiver and experimenter watched the videos together and rewatched as needed. During this time, the experimenter reminded the caregiver of the goals and procedures of the study. The caregiver then completed the social validity questionnaire independently. The experimenter used a random generator to select the videos. Three different types of video recordings were included for each participant: (1) one baseline session with toys and real objects for each play target, (2) one training session with real objects before mastery, and (3) one session showing learned‐combinations play actions for each target.

### Interobserver agreement and procedural fidelity

A second independent, trained observer also recorded data for at least 62% of randomly selected sessions across baseline with toys, generalization probes with toys, and real object baseline and teaching. Observers were trained on the different targets of learned‐combinations play actions and vocalizations using instruction, modeling, and role‐play practice with feedback on data collection. An agreement was scored if both observers recorded a response as correct or incorrect for a given trial. A disagreement was scored if one observer scored correct and the other observer scored incorrect for a given trial. Interobserver agreement was calculated by dividing the total agreements by the total number of agreements and disagreements and multiplying by 100. For Heidi and Roy, interobserver agreement was 100% for generalization probes with toys and 100% for real object teaching. Belle's interobserver agreement was 100% for generalization probes with toys and a mean of 99.56% (range: 96%–100%) for real object teaching.

Procedural fidelity data were collected during at least 62% of sessions for each participant to assess accuracy of implementation of toy probes and real object sessions. For baseline and generalization probes with toys, procedural fidelity data were assessed for correct stimulus presentation, instruction delivery, time provided, and absence of directions or programmed consequences. For real object sessions, procedural fidelity data were assessed for correct stimuli and instruction, time provided, least‐to‐most prompting provided as needed, and correct programmed consequences. Each trial was scored as incorrectly or correctly administered. The number of correct trials was divided by the total number of trials (correct and incorrect) and then multiplied by 100. Heidi's procedural fidelity was 100% for toy probes and 100% for real object sessions. Roy's procedural fidelity was 100% for toy probes and a mean of 99.78% (range: 99%–100%) for real object sessions. Belle's procedural fidelity was 100% for toy probes and a mean of 99.67% (range: 99%–100%) for real object sessions.

## RESULTS

**FIGURE 1:**
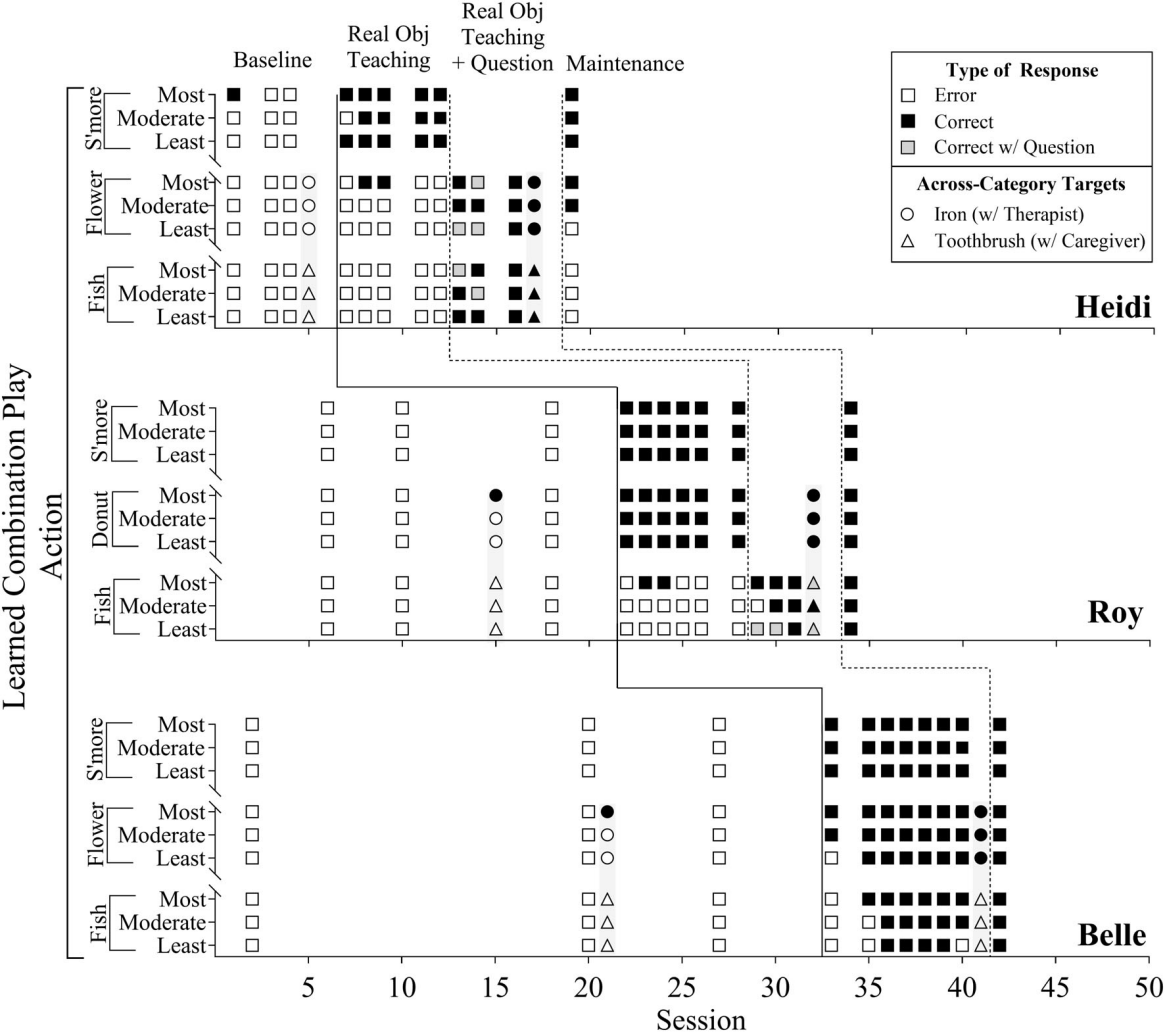
Learned‐combinations play actions. The squares depict the occurrence (i.e., dark symbols) and nonoccurrence (i.e., white symbols) of learned‐combinations play actions per toy set. The circles and triangles on the gray bars depict the pre‐ and posttests of the across‐category targets that the participants' therapists and caregivers completed with them during real object teaching. Obj. = object.

**FIGURE 2:**
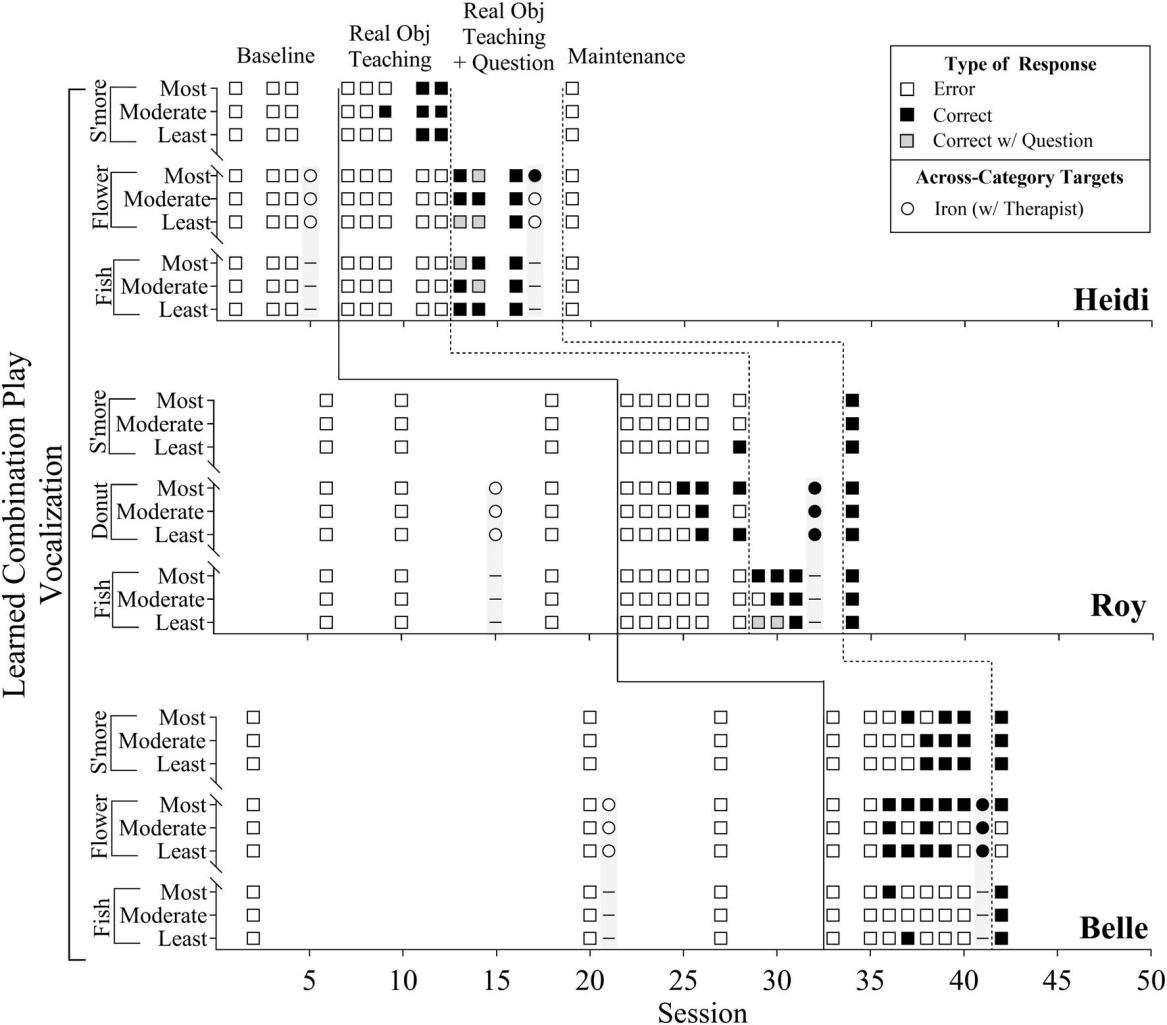
Learned‐combinations play vocalizations. The squares depict the occurrence (i.e., dark symbols) and nonoccurrence (i.e., white symbols) of learned‐combinations play vocalizations per toy set. The circles on the gray bars depict the pre‐ and posttests of the across‐category targets that the participants' therapists completed with them during real object teaching. The dashed lines on the gray bars indicate no opportunities for play vocalizations with the toothbrush toy due to the lack of a corresponding vocalization. Obj. = object.

Figure 1 shows the occurrence and nonoccurrence of learned‐combinations play actions per play target across sessions, and Figure 2 shows the occurrence and nonoccurrence of learned‐combinations play vocalizations per play target across sessions. The *y*‐axis represents the play targets for each participant and identifies different exemplars per play target (e.g., s'mores toys most similar to real objects). The shading of each symbol corresponds to the type of learned combinations the participant emitted during play, with darker symbols indicating correct responses and white symbols indicating errors. This shading shows whether the participant met the mastery criterion of two correct responses out of three exemplars per play target (i.e., two darker symbols per target). Supporting Information I and J include line graphs showing the percentage of learned‐combinations play actions and vocalizations.

In Figure 1, each participant's performance on learned‐combinations play actions is displayed across sessions. During baseline, Heidi demonstrated low levels of the play actions (*M* = 3.66%, range: 0%–11.11%), with only one occurrence observed in her first session with the most similar s'more play target. Roy and Belle did not emit any play actions during baseline; however, they emitted one play action of the across‐category target. When real object teaching was introduced, the play actions increased across all participants. Each participant met mastery for specific play actions: Heidi met mastery primarily for one play target (i.e., s'more; *M* = 35.20%, range: 22.22%–44.44%), Roy met mastery for two play targets (i.e., s'more and donut; *M* = 70.67%, range: 66.67%–77.78%), and Belle met mastery for all play targets (i.e., flower, s'more, fish; *M* = 89%, range: 55.56%–100%). Following the introduction of the question (“Can you show me what else you can do with the toys?”), Heidi and Roy showed a rapid increase in the play actions for the targets they had not yet mastered. This resulted in Heidi meeting mastery for both the flower and fish play targets (100%) and Roy meeting mastery for the fish play target (*M* = 89%, range: 66.67%–100%). Note that the question was presented in five trials (55.56%) for Heidi and two trials (22.22%) for Roy. In the across‐category generalization probes, both Heidi and Roy's play actions increased from (0%) at pretest to (100%) at posttest, although Roy required the presentation of the question during two trials (33.33%). Belle's across‐category generalization probes increased from 0% to 50%. During the 1‐day maintenance probe, Heidi engaged in the play actions in more than half of the (55.56%) trials, whereas Roy and Belle maintained the play actions across all trials (100%).

In Figure 2, each participant's performance on learned‐combinations play vocalizations is displayed across sessions. During baseline, all three participants did not engage in the play vocalizations. When real object teaching was introduced, there was an increase in the play vocalizations for all participants. Both Heidi and Roy's play vocalizations increased (*M* = 15.40%, range: 0%–33.33%), primarily with the s'more and donut targets, respectively. Belle showed an increase in the play vocalizations (*M* = 34.86%, range: 0%–55.56%) primarily with the s'more and flower targets. Following the introduction of the question to Heidi's and Roy's unmastered targets, there was a rapid increase of the play vocalizations. Heidi's and Roy's play vocalizations aligned with the observed pattern in their play actions. In the across‐category generalization probes, Roy and Belle's play vocalizations increased for the iron play target from pretest (0%) to posttest (100%). Heidi's play vocalization increased for only one of the three trials (33.33%). Additionally, it should be noted that there were no opportunities to emit the play vocalizations with the toothbrush toy, as there was no corresponding vocalization for it. During the 1‐day maintenance probe, the play vocalizations were observed at varying levels. Heidi did not maintain any play vocalizations, Roy maintained the play vocalizations in all trials (100%), and Belle maintained the play vocalizations in most trials (77.78%).

The results of the preintervention social validity assessment showed a mean rating of 4.89 (range: 4–5; 1 = *strongly disagree*, 5 = *strongly agree*). All caregivers reported high levels of agreement to the statements indicating that the goals and procedures were important and acceptable. The only statement that did not receive a score of 5 was “I find it important that my child experience real activities that they can demonstrate through play.” Additionally, the results of the postintervention social validity assessment showed a mean rating of 4.95 (range: 4–5). All caregivers reported high levels of agreement to the statements in the postintervention social validity assessment of the goals and the procedures. Comments included, “Learning from real world experiences is a skill my child can use as she gets older.”

## DISCUSSION

We evaluated the effects of teaching actions with real objects on generalized toy play with children with ASD. All participants mastered the learned‐combinations play actions, demonstrating that generalized play can be programmed by teaching with real objects, replicating Lee et al. (2021). This study extends Lee et al. by (a) not providing contrived reinforcement after play responses, (b) targeting nonverbal pretend play behavior, and (c) incorporating a general‐case analysis (Haring et al., 1985). Although previous interventions have taught pretend play actions to children with ASD (e.g., Barton & Wolery, 2010; MacDonald et al., 2009; MacManus et al., 2015), the resulting play behaviors may differ in function from those commonly observed in neurotypical children (e.g., Luckett et al., 2007). That is, even though participants engaged in play responses, these responses were directly reinforced rather than reflecting real‐life experiences. This study extends previous research by evaluating the emergence of learned‐combinations play actions with children with ASD, similar to those of their neurotypical peers.

Heidi mastered one play target, Roy two, and Belle all three through real object teaching alone. Heidi and Roy met the remaining targets when the question (i.e., “Can you show me what else you can do with the toys?”) was introduced with the real object teaching. The nature of the tasks likely influenced these outcomes. The s'more and donut play targets for Heidi and Roy did not require the addition of a question. Play targets like assembling a s'more and decorating a donut, which require specific sequences, can be viewed as behavior chains (Cooper et al., 2020) that yield a terminal outcome. In contrast, actions with the flower and the fish targets did not require a specific order. This suggests that generalized learned‐combinations play involving behavior chains may be easier to acquire and could serve as an effective starting point in teaching learned‐combinations play. However, further research is needed to determine whether there are advantages to teaching behavior chains first.

Automatic reinforcement and motivating operations may have influenced mastery of some targets with only real object teaching, but others required the question. Automatic reinforcement strengthens behavior without the mediation of another person, resulting from behavior that directly affects the individual's body or environment (Vaughan & Michael, 1982). Schlinger (1995) illustrated this with rhythmic behaviors exhibited by newborns (e.g., banging hands on a surface), which may be described as “spontaneous.” He suggested that the reinforcing consequences of these behaviors (e.g., auditory stimulation from banging) may not be immediately obvious. In the current study, Roy engaged with the most similar fish toy by tapping the fishbowl and pouring food, which involved a moving toy fish in a water‐filled bowl. He tapped the fishbowl when the toy fish was stationary, potentially indicating the presence of an establishing operation for the toy fish to move in the water. Additionally, Roy may have accessed automatic reinforcement when the toy fish moved after tapping the fishbowl. Similarly, Heidi engaged only with most similar flower target (i.e., smelling and putting the flower in a vase), likely due to its scent. However, this behavior did not persist, possibly indicating a decrease in the reinforcer's value for Heidi. Belle, who mastered all play targets with real object teaching, also initially engaged with the most similar fish and flower toys. These play actions with the toys that were most similar to the real object may reflect sensory properties (e.g., visual feedback of the toy fish moving after tapping the fishbowl) of real objects that could function as automatic reinforcement. If sensory features enhance learning of generalized play, future research could explore selecting toys with salient sensory properties to increase play with children with ASD. Another variable that might influence play is a child's history or preference for a toy in other contexts, such as a child who enjoys Lincoln Logs being more likely to engage with similar wooden building sets during play.

The Developmental Play Assessment for Practitioners (Lifter et al., 2022) was used to select play targets (Level 9, Varied Action Sequences) that matched the emerging developmental status of all participants, which replicated the procedure that has been used in prior research (e.g., Agana et al., 2024; Kim, 2016; Lifter et al., 1993; Pane et al., 2022; Scheflen et al., 2012). Targeting these play targets may have led to faster acquisition than if targets were not matched to development, as demonstrated in previous studies (e.g., Lifter et al., 1993). Our findings mirrored those of Agana et al. (2024) and Pane et al. (2022) showing increased play postintervention, without contrived consequences (e.g., tangible reinforcers). It is possible that contrived consequences are not always necessary in increasing development‐matched play, which may be more likely to produce automatic reinforcement due to an establishing operation for specific types of sensory stimulation that changes throughout the development of play (Pane et al., 2022). Future research should further explore the developmental progression of play, as described by developmental psychologists, through the lens of principles of behavior.

We used a general‐case analysis (Horner et al., 1984) to identify real objects and toys considering critical and noncritical features that increase the likelihood of responses occurring under appropriate stimulus control. Thus, the toys selected would serve as discriminative stimuli to engage in the actions taught with the real objects (Bijou, 1995). Identifying critical features can help researchers, clinicians, and caregivers select toys for programming generalization in play. We assessed the generalization of two types of across‐category targets: (1) actions taught by a familiar therapist and (2) actions already part of the participant's daily routine with caregivers. The results showed two types of generalization. First, all participants demonstrated learned‐combinations play from actions taught by their therapists, indicating generalization across people. Second, Heidi and Roy showed learned combinations‐play from actions they were already engaging in at home, suggesting that they began to show a generalized repertoire of their development‐matched play target with other toys. However, these interpretations are limited as our study used only one set of targets and lacked repeated measures. Future studies could evaluate across‐category generalization and the generalized repertoire of development‐matched play targets using more targets and repeated measures. Belle did not show generalization to toys reflecting activities with her caregivers, possibly due to differences in stimulus control and generalization (e.g., variations between home and clinic settings; the time elapsed between real object actions and toy probes; the presence of the experimenter). Future studies could include across‐category targets to clarify these variables and improve generalization programming.

The social validity of the goals, procedures, and outcomes was assessed via caregiver interviews and questionnaires pre‐ and postintervention, addressing a gap in the literature where social validity is often conducted only postintervention (Leif et al., 2024). Overall, high social validity was indicated, but procedures involving real‐life play actions received slightly lower scores. Because caregiver involvement was limited, the procedures might not have been deemed as important. Future studies could involve caregivers as behavior‐change agents and further assess the social validity of the procedures. The study's procedures may also be socially valid as they offer caregivers opportunities to foster real‐life interactions with their children. A practical implication is the value of involving natural change agents (e.g., caregivers) in real‐life activities to promote pretend play. Although participants met mastery criteria for primary targets, future research could present real objects and toys concurrently. For example, a child might play with a cooking toy set while observing caregivers cook. Future research could investigate whether children are more likely to imitate real actions if similar toys and materials are available during those activities. Additionally, future research could further assess social validity by conducting a preference assessment of toys to determine participants' preferences relative to other items.

Our study has several considerations and limitations. First, it was conducted in an analog setting with sequential presentation of objects and trial‐by‐trial data collection. Future studies could adopt more naturalistic methods, presenting toys simultaneously and collecting frequency data to better reflect typical play environments. Second, our intervention taught actions with real objects followed by immediate toy probes, as our purpose was not to evaluate the timing between teaching and generalization probes. Our maintenance data showed that Roy and Belle maintained play actions during probes conducted 1 day after real object teaching, but the extent to which these play behaviors will occur outside session parameters remains unclear. Future research could examine longer intervals between real object teaching and toy probe sessions in more naturalistic play settings.

Third, participants met specific prerequisite skills (i.e., object imitation, perceptual‐based matching, Learned Combination of Objects), which may limit generality to children without these skills. Their mastery of Learned Combinations of Objects in the Developmental Play Assessment for Practitioners (Lifter et al., 2022) suggested readiness for engaging in play actions reflecting daily activities in their culture. However, given that participants mastered Learned Combinations of Objects (Level 6), the study focused on teaching Varied Action Sequences (Level 9). Varied Action Sequences, a higher play level, involve engaging in two or more related actions within a play activity. For example, a child might stir in a toy pot (Action 1) and then pour the contents into a toy bowl (Action 2). Varied Action Sequences expands on Learned Combinations of Objects by similarly using conventional toys but further requiring multiple steps within a play activity. Future studies could explore the efficacy of the method for children with ASD whose play is at an earlier developmental level than that of the participants in this study, particularly those who have not yet mastered Learned Combinations of Objects.

Fourth, the question “Can you show me what else you can do with the toys?” did not directly teach play actions with the toys but allowed for generalization assessment. Heidi and Roy may have engaged with the targets solely in response to the question, without prior real object teaching. Future research could isolate its effects by including it in all baseline sessions, regardless of play responses. However, this may limit the study's analysis of potential automatic reinforcement in a child's play, as the question could function as an instruction rather than an opportunity for spontaneous engagement. Although it did not directly prompt specific play actions in the way that prompts are typically used in play studies, the question could be conceptualized as a prompt. Instead, we see this question as similar to asking, “What is it?” to prompt a tact response from a child who did not do so spontaneously.

Fifth, the toy selection process warrants consideration. Although the general‐case analysis identified critical features of items (e.g., stem, petal structure of a flower), this was done solely by the first author. Future research could include interobserver agreement to identify critical and noncritical features of items. Additionally, activities were strategically varied across rooms (e.g., making s'mores in the kitchen, feeding fish in an office) to reduce the likelihood that generalization to the toys occurred because all the targets used were from the same setting (e.g., the kitchen). Consequently, we were unable to randomize the selection of toys as primary or across‐category targets. Future research could explore methods to allow randomization of primary and across‐category targets.

In conclusion, our study extended the literature by demonstrating the efficacy of a general‐case analysis and developmental play approach as well as teaching real‐life activities in improving pretend play in three children with ASD. These findings contribute to behavior analytic literature in seeking to bridge gaps in understanding the efficacy of programming for generalization and learning actions with real objects as well as demonstrating their influence on generalized play skill development for children with ASD. This study also replicates and extends research from other disciplines (e.g., Piaget, 1962), showing that children's pretend play reflects their daily activities while applying behavior analytic interpretations (Bijou, 1995; Schlinger, 1995) to these findings and offering a more unified theoretical approach to understanding the development of play for children with ASD.

## AUTHOR CONTRIBUTIONS

The first author led the conceptualization, methodology development, data curation, formal analysis, and investigation for the project. The first author also secured funding, managed project administration, and prepared the original draft of the manuscript. The second author contributed to the conceptualization and methodology and provided critical review and editing of the manuscript. The third author supported methodology development, provided essential resources, and offered supervision and mentorship throughout the project. The fourth and fifth authors contributed resources necessary for conducting the study.

## CONFLICT OF INTEREST STATEMENT

The authors declare that they have no conflicts of interest.

## ETHICS APPROVAL

Our research was reviewed and approved by Caldwell University's Institutional Review Board. Our research adheres to strict ethical standards, including obtaining informed consent from participants, ensuring confidentiality of data, protecting participants from harm, disclosing any conflicts of interest, accurately attributing authorship and contributions, maintaining originality and avoiding plagiarism, complying with ethical guidelines, and upholding data integrity.

## Supporting information


**Data S1:** Supporting Information

## Data Availability

The data that support the findings of this study are available from the corresponding author upon reasonable request.
