# Supplementary material for: An analysis of the generalization of pretend play from real objects to toys
Source: J Appl Behav Anal. 2025 Jun 10;58(3):478–89. doi: 10.1002/jaba.70017 (PMC12302316; doi:10.1002/jaba.70017)
Supplement: Supplementary file 1 — Data S1: Supporting Information [file JABA-58-478-s001.pdf]

## Supporting Information

### Supporting Information A

*Targets and Stimuli for Roy*

| Stimulus, Action, Vocalization | Target 1 | Target 2 | Target 3 | Across Category (Therapist) | Across Category (Caregiver) |
|---|---|---|---|---|---|
| Stimuli | Marshmallow, graham crackers, chocolate, stick | Donut base, icing, sprinkle | Fish, fishbowl, fish food container | Iron, iron board, clothing item, spray bottle | Toothbrush, cup |
| Action | (1) Put marshmallow on top of chocolate<br>(2) Put cracker on top of marshmallow | (1) Put donut on icing<br>(2) Put sprinkle on donut | (1) Put finger on fishbowl<br>(2) Put fish food above fishbowl | (1) Press spray bottle to clothing item<br>(2) Move iron side to side on clothing item | (1) Put toothbrush to teeth<br>(2) Put cup to mouth |
| Vocalization | (1) “Marsh-mallow”<br>(2) “Yummy!” | (1) “Glaze time”<br>(2) “Now sprinkle!” | (1) “Hi, fishie!”<br>(2) “Eat some food” | (1) “Spray it”<br>(2) “Then smooth” | N/A |

*Note*: The targeted play category was Varied Action Sequences, which consists of acting out two or more different play actions. N/A = Not applicable; the target was not included for that condition.

**Supporting Information B**

*Targets and Stimuli for Belle*

| **Stimulus, Action, Vocalization** | **Target 1** | **Target 2** | **Target 3** | **Across Category (Therapist)** | **Across Category (Caregiver)** |
|---|---|---|---|---|---|
| Stimuli | Marshmallow, graham crackers, chocolate, stick | Flower, vase | Fish, fishbowl, fish food container | Iron, iron board, clothing item | Toothbrush, cup |
| Action | (1) Put marshmallow on top of chocolate<br>(2) Put cracker on top of marshmallow | (1) Touch flower to nose<br>(2) Put flower in vase | (1) Put finger on fishbowl<br>(2) Put fish food above fishbowl | (1) Turn dial on iron<br>(2) Move iron side to side on clothing item | (1) Put toothbrush to teeth<br>(2) Put cup to mouth |
| Vocalization | (1) "Stack"<br>(2) "Squish!" | (1) "Mmmmm"<br>(2) "Nice" | (1) "Fish!"<br>(2) "Eat" | (1) "Hot"<br>(2) "Smooth" | N/A |

*Note.* The targeted play category was *Varied Action Sequences*, which consists of acting out two or more different play actions. N/A = Not applicable.

**Supporting Information C**

*S'more Toy Exemplars*

| Features | Stimulus | Most Similar | Moderately Similar | Least Similar |
|---|---|---|---|---|
| Critical Features | *Marshmallow*: short cylinder shape<br><br>*Cracker*: four-sided, light brown<br><br>*Chocolate:* dark brown<br><br>*Stick:* long, skinny | All critical features included | | |
| Non-Critical Features | *Marshmallow*: size, color<br><br>*Cracker*: size, shade of brown<br><br>*Chocolate:* size<br><br>*Stick:* size | Non-critical features varied | | |
| Common Features | *Marshmallow*: white, soft, fluffy | *Marshmallow*: white, soft, fluffy | *Marshmallow*: white, soft | *Marshmallow*: soft |
| | *Cracker*: light brown | *Cracker*: light brown | *Cracker*: light brown | *Cracker*: light brown |
| | *Chocolate:* dark brown, dense | *Chocolate:* dark brown, dense | *Chocolate:* dark brown | *Chocolate:* dark brown |
| | *Stick:* light brown | *Stick:* light brown | *Stick:* light brown | *Stick:* light brown |

*Note*: The common features were systematically selected to closely resemble the most common characteristics of real objects depicted by similar toys. Conversely, moderately and less similar toys showed fewer common features that represent the real objects.

**Supporting Information D**

*Fish Toy Exemplars*

| Features | Stimulus | Most Similar | Moderately similar | Least Similar |
|---|---|---|---|---|
| Critical features | *Fish*: oval-shaped body, triangular-shaped tail<br><br>*Fishbowl*: opening or mouth, base or foot<br><br>*Fish food container*: cylinder shaped | All critical features included | | |
| Non-Critical features | *Fish*: color, size<br><br>*Fishbowl*: color, design, size<br><br>*Fish food container*: color, design, size | Non-critical features varied | | |
| Common features | *Fish*: eyes, mouth, fin, swims | *Fish*: eyes, mouth, fin, swims | *Fish*: eyes, mouth, fin | *Fish*: eyes, mouth, fin |
| | *Fishbowl*: water, hard glass/plastic | *Fishbowl*: water, hard plastic | *Fishbowl*: hard plastic | *Fishbowl*: n/a |
| | *Fish food container*: food inside | *Fish food container*: food inside | *Fish food container*: none | *Fish food container*: none |

*Note.* The common features were systematically selected to closely resemble the most common characteristics of real objects depicted by similar toys. Conversely, moderately and less similar toys showed fewer common features that represent the real objects.

**Supporting Information E**

*Donut Toy Exemplars*

| Features | Stimulus | Most Similar | Moderately Similar | Least Similar |
|---|---|---|---|---|
| Critical features | *Donut base*: round shape, brown<br><br>*Icing*: smooth, liquid texture<br><br>*Sprinkles:* cylinder shape | All critical features included | | |
| Non-Critical features | *Donut base*: size, texture, shade of brown<br><br>*Icing*: color, thickness<br><br>*Sprinkles:* number of pieces, color, size | Non-critical features varied | | |
| Common features | *Donut base*: fluffy, soft, light brown, ring-shaped hole in the middle, palm-sized | *Donut base*: fluffy, soft, light brown, ring-shaped hole in the middle, palm sized | *Donut base*: light brown, ring-shaped hole in the middle | *Donut base*: ring-shaped hole in the middle |
| | *Icing*: white | *Icing*: white | *Icing*: white | *Icing*: white |
| | *Sprinkles:* group of pieces, multi-colored, three-dimensional | *Sprinkles:* group of pieces, multi-colored, three-dimensional | *Sprinkles:* group of pieces, multi-colored, three-dimensional | *Sprinkles:* group of pieces |

*Note.* The common features were systematically selected to closely resemble the most common characteristics of real objects depicted by similar toys. Conversely, moderately and less similar toys showed fewer common features that represent the real objects.

**Supporting Information F**

*Iron Toy Exemplars*

| Features | Stimulus | Most Similar | Moderately Similar | Least Similar |
|---|---|---|---|---|
| Critical features | *Iron*: triangular base, handle at top<br><br>*Iron board*: rectangular board, triangular pane on one end of board<br><br>*Clothing item:* flat, soft texture | All critical features included | | |
| Non-Critical features | *Iron*: weight, color, size<br><br>*Iron board*: size<br><br>*Clothing item:* size, material | Non-critical features varied | | |
| Common features | *Iron*: circular dial, silver bottom | *Iron*: circular dial, silver bottom | *Iron*: circular dial, silver bottom | *Iron*: circular dial |
| | *Iron board*: soft cover, legs for standing | *Iron board*: soft cover, legs for standing | *Iron board*: legs for standing | *Iron board*: none |
| | *Clothing item:* smooth | *Clothing item:* smooth | *Clothing item:* smooth | *Clothing item:* smooth |

*Note.* The common features were systematically selected to closely resemble the most common characteristics of real objects depicted by similar toys. Conversely, moderately and less similar toys showed fewer common features that represent the real objects.

**Supporting Information G**

*Toothbrush Toy Exemplars*

| Features | Stimulus | Most Similar | Moderately Similar | Least Similar |
|---|---|---|---|---|
| Critical features | *Toothbrush*: rectangular-shaped stick at bottom, rectangular block at top<br><br>*Cup*: cylinder shape, opening or mouth, base or foot | All critical features included | | |
| Non-Critical features | *Toothbrush*: color, pattern, texture, size<br><br>*Cup*: color, design, size | Non-critical features varied | | |
| Common features | *Toothbrush*: toothpaste, bristle texture | *Toothbrush*: toothpaste, bristle texture | *Toothbrush*: bristle texture | *Toothbrush*: n/a |
| | *Cup*: water | *Cup*: water | *Cup*: none | *Cup*: none |

*Note.* The common features were systematically selected to closely resemble the most common characteristics of real objects depicted by similar toys. Conversely, moderately and less similar toys showed fewer common features that represent the real objects.

**Supporting Information H**

*Social Validity Assessment*

| **Pre-intervention Assessment: Rating Scale** |
|---|
| It is important to me that my child's play continue to develop. |
| I value that my child can engage in play that reflects their environment. |
| I find it important that my child experience real activities that they can demonstrate through play. For example, if my child helps with cooking, I would find joy in seeing them do those actions with a toy cooking set. |
| **Post-intervention Assessment: Rating Scale** |
| The goal of this study was important for my child. |
| The procedures used to teach play to my child were valuable. |
| The techniques used in this study for promoting my child's play were feasible and practical. |
| I find it more valuable that my child's play developed using *this* procedure than teaching them how to *directly* engage with the toys. |
| The strategies used in the study can be used in everyday routines or activities. |
| My child engaged with the toys similar to how they engaged with the real objects. |
| I would recommend similar interventions to other caregivers or clinicians of children with autism. |
| **Open-ended question** |
| Were there any aspects of the study procedures that you found particularly beneficial for your child or your family? |
| Were there any aspects of the study procedures that you found particularly challenging for your child or your family? |
| Can you describe any instances where you observed your child showing enjoyment or enthusiasm during the study sessions? |

*Note*: Respondents were asked to rate questions with numbers (1 = *Strongly Disagree*, 2 = *Disagree*, 3 *Neutral*, 4 = *Agree*, 5 = *Strongly Agree*) that best reflected their experience during the study.

**Supporting Information I**

*Percentage of Trials with Learned Combinations of Play Actions*

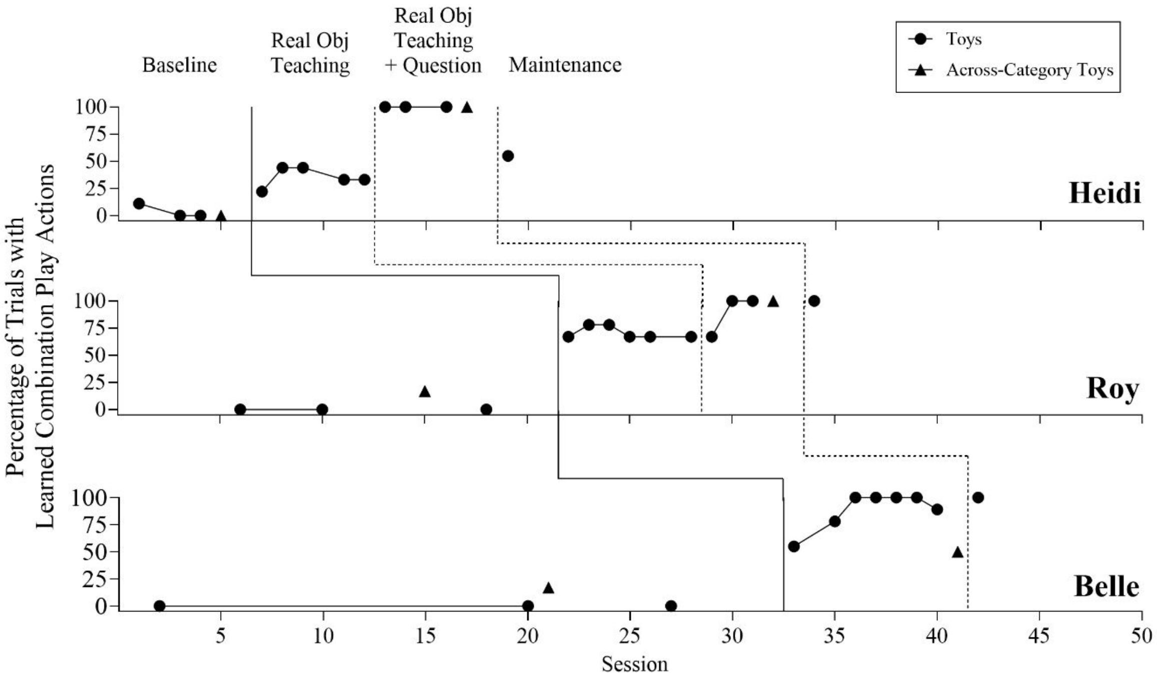


*Note*: Obj. = object.

**Supporting Information J**

*Percentage of Trials with Learned Combinations of Play Vocalizations*

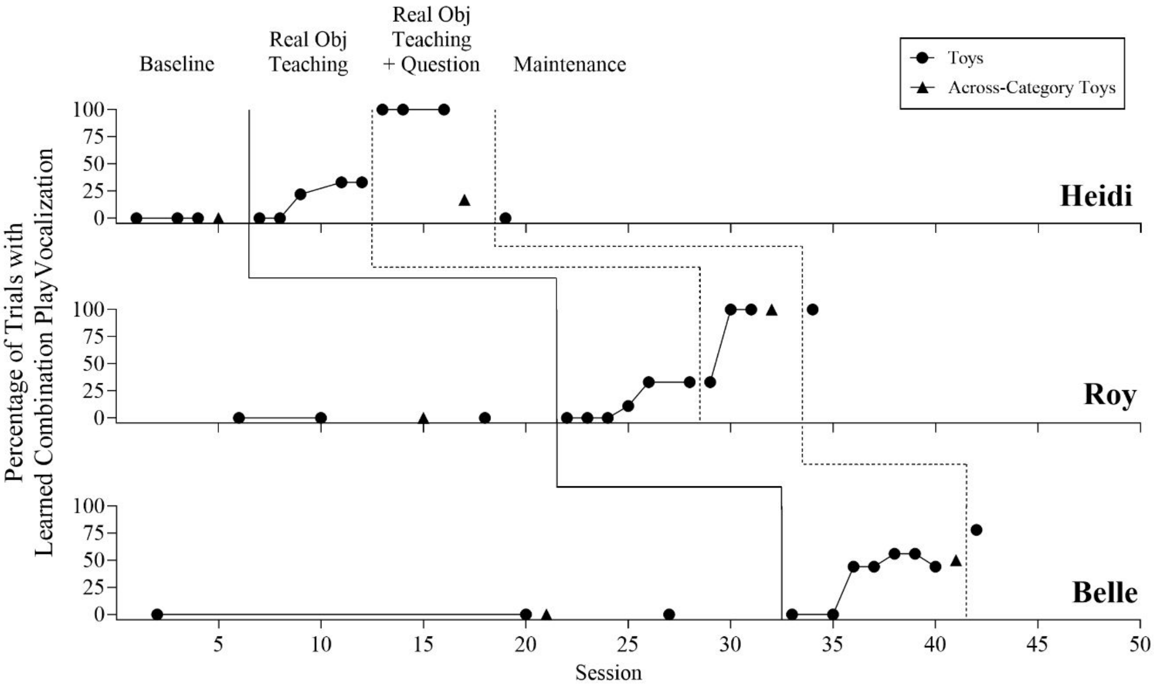


*Note*: Obj. = object.
